# Rapid Fabrication of Large-Area Anti-Reflective Microholes Using MHz Burst Mode Femtosecond Laser Bessel Beams

**DOI:** 10.3390/nano15221726

**Published:** 2025-11-15

**Authors:** Yulong Ding, Cong Wang, Zheng Gao, Xiang Jiang, Shiyu Wang, Xianshi Jia, Linpeng Liu, Ji’an Duan

**Affiliations:** State Key Laboratory of Precision Manufacturing for Extreme Service Performance, College of Mechanical and Electrical Engineering, Central South University, Changsha 410083, China; dingyulong@csu.edu.cn (Y.D.); 233712195@csu.edu.cn (Z.G.); 243706046@csu.edu.cn (X.J.); 243712181@csu.edu.cn (S.W.); 221026@csu.edu.cn (X.J.); linpengliu@csu.edu.cn (L.L.); duanjian@csu.edu.cn (J.D.)

**Keywords:** femtosecond laser, functional microstructure, anti-reflection, large area, high-consistency

## Abstract

Femtosecond laser has been widely utilized in functional microstructural surfaces for applications such as anti-reflection, radiative cooling, and self-cleaning. However, achieving high-efficiency manufacturing of high-consistency functional microstructures (with feature sizes ~1 μm) over large areas remains a challenge. Here, we report a femtosecond laser temporal and spatial modulation technique for fabricating large-area anti-reflective microholes on magnesium fluoride (MgF_2_) windows. The beam was transformed into a Bessel beam to extend the Rayleigh length, enabling the fabrication of microhole arrays with sub-micron precision and surface roughness variations within 10 nm over a 6 μm focal position shift range (5–11 μm). By modulating MHz burst pulses, the aspect ratio of the microholes was increased from 0.3 to 0.7 without compromising a processing speed of 10,000 holes per second. As a proof of concept, large-area anti-reflective microholes were fabricated on a 20 mm × 20 mm surface of the MgF_2_ window, forming a nanoscale refractive index gradient layer and achieving a transmittance increase to over 98%. This method provides a feasible solution for the efficient and high-consistency manufacturing of functional microstructures over large areas.

## 1. Introduction

Femtosecond laser processing of large-area micro- and nano-structures has demonstrated outstanding functionality in the field of functional surfaces, such as radiation cooling [1,2], self-cleaning [3,4], and anti-reflective properties [5,6]. Typically, functional surfaces are composed of tens of thousands of micro- and nano-structural units covering areas of several centimeters [4,7]. For functional microstructures with feature sizes in the range of hundreds of micrometers, femtosecond laser processing systems, such as scanning galvanometers, can achieve high efficiency and consistency in manufacturing [8,9,10,11]. However, as the feature sizes of the functional microstructures approach 1 μm, significant challenges arise in the large-area application of functional surfaces. On one hand, reducing the feature size of the microstructures by half results in a fourfold increase in the number of structural units that need to be fabricated. The quantity of microstructural units may increase to millions or even billions, necessitating substantial time and resources for production [12]. On the other hand, achieving high-precision processing requires the use of high-magnification objectives to reduce the beam diameter, which significantly decreases the Rayleigh length of the focused beam [13,14]. Consequently, ensuring that the laser focus consistently remains at the same position on the material surface during large-scale fabrication becomes difficult [15,16]. This inconsistency in the manufactured microstructures adversely affects the performance of the functional surfaces [17].

Recently, inspired by the compound eye structures of insects (Figure S1a, Supporting Information), the fabrication of anti-reflective microstructures on infrared window surfaces has become a major focus of research [18,19,20]. The principle involves constructing periodic microstructural arrays on the material surface to form a nanoscale refractive index gradient layer (Figure S1b, Supporting Information), which can effectively reduce light loss on the material surface [21,22,23,24,25,26,27,28]. Traditionally, anti-reflective properties are achieved by depositing single or multilayer optical coatings [29]. While these coatings are highly effective in specific wavelength ranges, their performance can degrade under extreme environmental conditions, such as temperature fluctuations, mechanical abrasion, and moisture exposure, due to issues like thermal mismatch and poor adhesion [12]. In contrast, anti-reflective microstructures are directly fabricated on the surface of the window material. This monolithic integration offers superior mechanical robustness, environmental durability, and the potential for broadband performance [19].

As a critical component of infrared thermal imaging systems, infrared windows are primarily used for the transmission of infrared signals [30]. To achieve practical applications of anti-reflective windows in thermal imaging, high-performance anti-reflective microstructures need to be fabricated over large areas. Wang et al. demonstrated the fabrication of approximately 30 million structural units within 50 min using femtosecond laser single-pulse processing of microholes [31]. However, due to the limited energy of a single pulse, the aspect ratio of the fabricated anti-reflective microholes was only 0.2. According to equivalent medium theory and classical thin-film theory, the depth of microholes is closely related to the anti-reflective effect [12,32]. Enhancing the anti-reflective performance necessitates increasing the depth of the structures. Yun et al. proposed a focal multiplexing technique to process microgrooves, achieving an aspect ratio exceeding 1:1 [16]. Although this improved the aspect ratio, the scanning speed was low and required multiple scans, significantly increasing fabrication time. Moreover, the Gaussian beams used in these studies had relatively short Rayleigh lengths, with effective processing ranges below 20 μm [15]. As the defocus amount changed, noticeable variations occurred in the contour parameters of the fabricated microstructures. The presence of these challenges continues to hinder the efficient and high-performance fabrication of anti-reflective windows.

Here, a method is proposed for the rapid fabrication of large-area anti-reflective microholes on the surface of magnesium fluoride (MgF_2_) windows using MHz burst mode femtosecond laser Bessel beams. Utilizing a repetition rate of 10 kHz and four burst sub-pulses for rapid scanning, the aspect ratio of the microholes was increased to approximately 0.7 without compromising a scanning speed of 20 mm/s. The transformation of Gaussian beams into Bessel beams was employed to enhance the Rayleigh length, resulting in processing errors of less than 0.1 μm and surface roughness variance below 10 nm, even with a variation in the focal position of 6 μm. By integrating these two techniques, bow-tie scanning was performed at a rate of 10,000 holes per second over a 20 mm × 20 mm area of the MgF_2_ window, successfully fabricating large-area anti-reflective microholes and increasing the transmittance to over 98%.

## 2. Experiment

**Figure 1:**
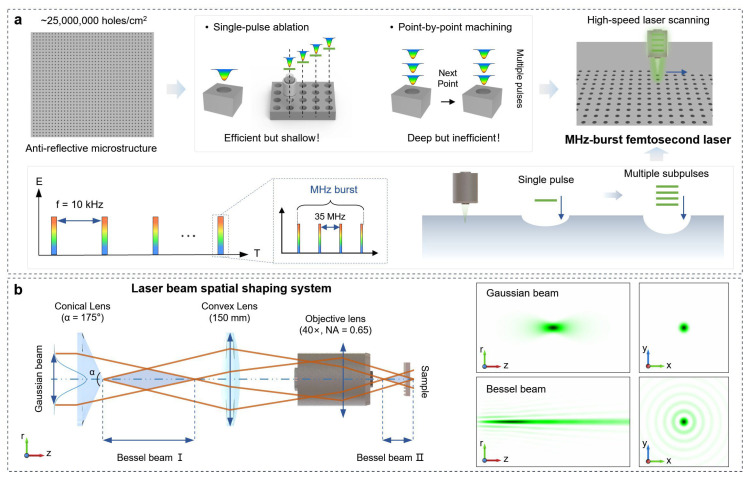
(**a**) Schematic of the femtosecond laser MHz burst mode scanning method; (**b**) Schematic of the optical path for transforming a Gaussian beam into a Bessel beam.

The experimental samples are double-sided polished magnesium fluoride (MgF_2_) windows, measuring 20 mm × 20 mm, which were securely fixed onto a three-axis motion platform (HS-DX3-V200, Hostechn, Changsha, China) using a custom-made fixture. During the fabrication process, the laser focus was maintained in a fixed position, while the three-axis motion platform facilitated bow-tie scanning of the sample at a speed of 20 mm/s to process the microholes. The laser source used was a high-power ultrafast femtosecond laser (HR-300-1, Ultron Photonics, Hangzhou, China) with an output wavelength of 515 nm, a pulse width of 844 fs, and a repetition rate of 10 kHz. The burst mode of the laser was adjustable to output pulse sequences containing between 1 and 10 sub-pulses (Figure 1a). The beam shaping system utilized an axicon with a cone apex angle of 175°, while the beam focusing system employed a combination of a convex lens (150 mm) and an objective lens (NA = 0.65, 40×) (Figure 1b). The morphology of the microholes was evaluated using a laser scanning confocal microscope (LSCM, LSM700, Carl Zeiss, Oberkochen, Germany) and a field-emission scanning electron microscope (SEM, JSM-7900F, JEOL, Tokyo, Japan). The transmittance of the samples was evaluated using a Fourier transform infrared spectrometer (Nicolet iS50, Thermo Fisher, Waltham, USA). The measurements were conducted in transmission mode with the following parameters: a spectral resolution of 4 cm^−1^ over the wavenumber range of 4000–400 cm^−1^, and 32 scans were accumulated and averaged for both the background (open beam) and the sample to ensure a high signal-to-noise ratio. The incident beam spot size on the sample was approximately 10 mm in diameter. The transmittance spectrum was automatically calculated by the instrument’s software (OMNIC, Thermo Scientific, Waltham, USA). The spectral data were then converted from wavenumber (σ, in cm^−1^) to wavelength (λ, in μm) for analysis and plotting using the equation [33,34]:

(1) $$\lambda =\frac{10,000}{\sigma }$$

For the simulation process, a light field simulation model was constructed using Finite-difference time domain (FDTD) methods [35]. The simulation setup primarily included boundary conditions, excitation sources, substrate materials, and monitors [36]. Perfectly matched layer (PML) boundary conditions were applied in the z-direction to eliminate reflections of plane waves from the computational domain. Periodic boundary conditions were established in the x and y directions to ensure the periodic arrangement of microstructures while reducing simulation time. A plane wave was selected as the excitation source (3–5 μm). Although an ideal plane wave is considered to extend infinitely in its wavefront. However, in practical scenarios, light waves can be approximated as plane waves. Based on this approximation, plane waves are often chosen as the incident light source in optical simulations to simplify the analysis of optical phenomena. This approach preserves key optical characteristics while reducing computational complexity, enhancing the efficiency and convenience of the research process. The monitor selected was a field distribution monitor, which allows for real-time observation of electromagnetic field distributions and facilitates analysis and understanding through visual representation and flexible parameter settings.

## 3. Results and Discussion

To investigate the influence of microstructural size parameters on the anti-reflective performance of MgF_2_ window surfaces, a study was conducted using equivalent medium theory (EMT) and FDTD simulation methods [32]. Initially, the periodicity and depth parameters of the microstructures were preliminarily designed using equivalent medium theory:

(2) $$\frac{p}{\lambda }=\frac{1}{n_{2}+n_{1}\sin\theta_{max}}$$

(3) $$d_{min}=\frac{\lambda }{4\sqrt{n_{1}n_{2}}}$$

where p represents the periodicity of the microstructures, $d_{min}$ denotes the minimum depth for optimal anti-reflective performance, $n_{1}=1$ is the refractive index of air, $n_{2}=1.38$ is the refractive index of MgF_2_, λ is the wavelength of the incident light, and $\theta_{max}=90{}^{\circ}$ indicates the maximum angle of incidence. The MgF_2_ window primarily serves as a protective window for mid-wave infrared (3–5 μm) thermal imaging devices. Therefore, the periodicity of the anti-reflective microstructures is set to 2 μm, with the depth required to be at least greater than 0.64 μm.

**Figure 2:**
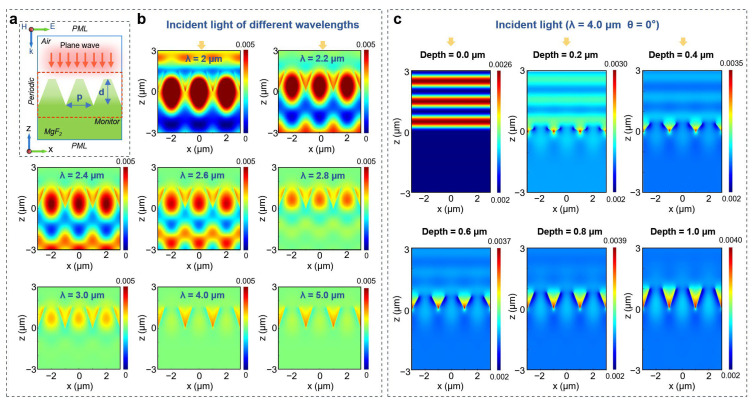
(**a**) Configuration of the FDTD simulation model; (**b**) Electric field distribution for different incident light wavelengths (2 μm, 2.2 μm, 2.4 μm, 2.6 μm, 2.8 μm, 3.0 μm, 4.0 μm, 5.0 μm) on the surface of the microstructures; (**c**) Electric field transmission of incident light at a wavelength of 4 μm on microstructures with different depths (0.0 μm, 0.2 μm, 0.4 μm, 0.6 μm, 0.8 μm, and 1.0 μm).

To more clearly elucidate the impact of microstructure periodicity and depth on anti-reflective performance, an electric field simulation model was constructed (Figure 2a). Figure 2b illustrates the distribution of electromagnetic field intensity during the interaction of incident waves at various wavelengths (including 2 μm, 2.2 μm, 2.4 μm, 2.6 μm, 2.8 μm, 3.0 μm, 4.0 μm, and 5.0 μm) under normal incidence conditions when the periodicity is set to 2 μm. Within the microstructure, the electromagnetic field intensity is significantly higher than in the surrounding areas. This phenomenon is primarily attributed to the effective capture of light waves by the subwavelength structures, resulting in multiple reflections and collisions of the light waves within the structures, which leads to a substantial increase in local electromagnetic field intensity [32,37]. This enhancement of the localized field strength can improve the capacity for light wave capture and effectively reduce reflection losses at the interface of the window material. When the incident wavelengths range from 2 μm to 2.6 μm, the similarity between the dimensions of the microstructures and the wavelengths results in significant scattering effects, causing the enhancement of higher-order diffraction and the weakening of zeroth-order diffraction. In this scenario, intense diffraction and scattering occur near the structures, leading to electromagnetic field intensities around the structures being markedly higher than at both ends of the propagation direction. Although this effect can reduce surface reflection, it alters the propagation direction of the light, thus not equating to an enhancement in infrared signal transmission capabilities (Figure S2a, Supporting Information). Starting from an incident wavelength of 2.8 μm, the electromagnetic field intensity around the structures approaches equivalence with that at both ends of the propagation direction, indicating a notable reduction in diffraction and scattering effects (Figure S2b, Supporting Information). However, localized field enhancement persists within the structures, demonstrating that the structures can effectively capture light waves, reduce surface reflection, and improve transmittance.

Figure 2c shows the distribution of electromagnetic field intensity during the interaction between microstructures with depths of 0.0 μm, 0.2 μm, 0.4 μm, 0.6 μm, 0.8 μm, and 1.0 μm and a plane wave with a wavelength of 4 μm. Specifically, the electromagnetic field intensity shows significant enhancement from a structure height of 0 μm to 0.6 μm, increasing from 0.0024 to 0.0037, with an enhancement of 0.0013. In contrast, from a structure height of 0.6 μm to 1.0 μm, the rate of increase in electromagnetic field intensity gradually slows, with an enhancement of only 0.0003, approaching stability. A stronger localized enhancement of the electromagnetic field intensity at the structure indicates an improved capacity for light wave capture and better anti-reflective performance. Additionally, alternating stripes of varying electromagnetic field intensity can be observed above the structures, primarily due to the presence of reflected light. When no structures are present, reflection losses are maximized, resulting in very pronounced stripes, with the electromagnetic field intensity entering the simulation area being higher than that leaving it. As the structure height increases, the stripes gradually fade, becoming almost nonexistent after 0.8 μm, indicating a reduction in reflection losses. Concurrently, the difference in electromagnetic field intensity between the entering and exiting regions of the simulation area decreases gradually. In the range of 0.6 μm to 1.0 μm, the electromagnetic field intensities at both ends trend towards equivalence, further demonstrating that the transmittance performance of the structure surface reaches an optimal level at this point. Therefore, the aforementioned simulation results provide clear theoretical guidance, indicating that to achieve high-performance anti-reflective properties in the mid-wave band, it is necessary to fabricate microholes with a period of less than 2.0 μm and a depth greater than 0.6 μm. However, traditional femtosecond laser Gaussian beams face significant challenges in efficiently manufacturing such microstructures over large areas, particularly in terms of processing efficiency and structural consistency. The disparity between this theoretical design and actual manufacturing capabilities has prompted the development of femtosecond laser spatiotemporal modulation techniques to meet the manufacturing demands.

**Figure 3:**
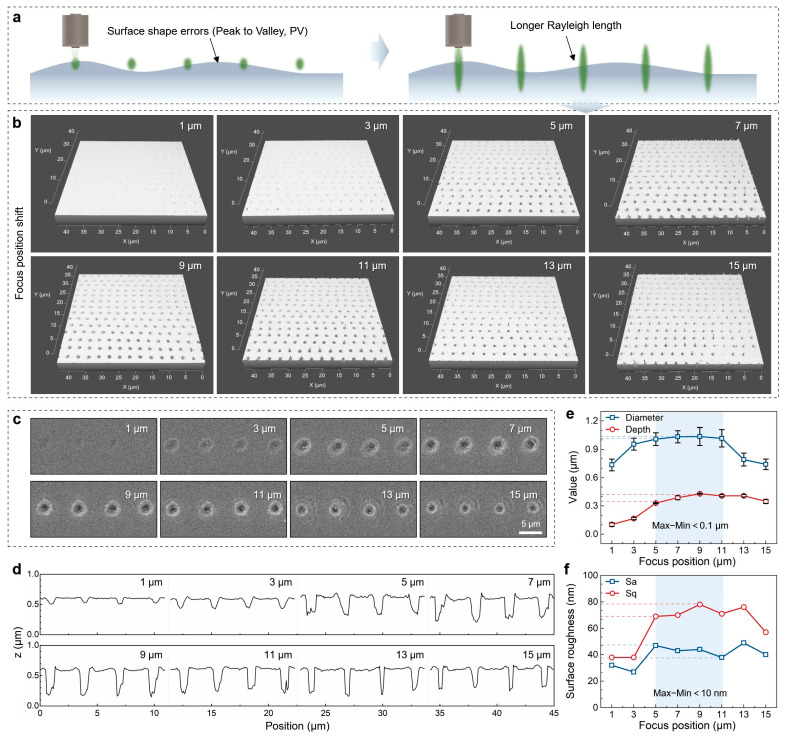
(**a**) Schematic of laser processing at different Rayleigh lengths of the focus on a surface with PV errors; (**b**–**d**) LSCM and SEM images of microholes processed with Bessel beams at focal positions of 1–15 μm; (**e**) Variation in the diameter and depth of the microholes with changes in the laser focal position; (**f**) Variation in the Sa and Sq of the microholes with changes in the laser focal position.

Currently, optical surface shape errors (Peak to Valley, PV) still exist on the window surface even after high-precision polishing [38]. To achieve high consistency in the manufacturing of microstructures, it is essential to enhance the Rayleigh length of the laser focus (Figure 3a). This is accomplished by transforming the Gaussian beam into a Bessel beam with a longer Rayleigh length [39]. Despite the focus drift caused by optical surface shape errors, the consistency of the manufactured microstructures remains high. Figure 3b–d illustrate the three-dimensional morphologies, two-dimensional morphologies, and central cross-sectional profiles of microholes at focal positions ranging from 1 μm to 15 μm. Good consistency is observed among the microholes fabricated at the same focal position. However, at focal positions of 13 μm and 15 μm, ablation of the substrate also occurred due to the increased energy of the Bessel side lobes (Figure 3c). Figure 3e depicts how the diameter and depth of the microholes vary with changes in the focal position. The results indicate that both the diameter and depth gradually increase from focal positions of 1 μm to 9 μm, and then decrease thereafter. Notably, the difference in diameter and depth of the microholes in the focal range of 5 μm to 11 μm remains less than 0.1 μm. Figure 3f shows the relationship between surface roughness (Sa and Sq) and the focal position, where the variation trend closely follows that of the microhole structural parameters. Within the focal range of 5–11 μm, the Sa and Sq values averaged 43 ± 3.2 nm and 72 ± 3.5 nm, respectively, demonstrating good stability. This suggests that by selecting an appropriate focal position, high-consistency microholes can still be fabricated, even in the presence of surface shape errors.

**Figure 4:**
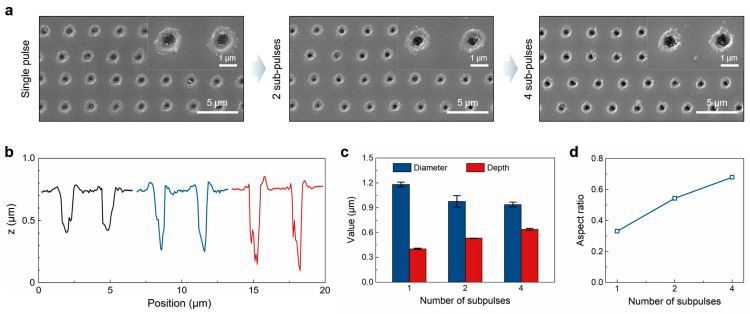
(**a**) SEM images of microholes fabricated with 1, 2, and 4 sub-pulses; (**b**) Central cross-sectional profiles of microholes fabricated with 1, 2, and 4 sub-pulses; (**c**,**d**) Diameter, depth, and aspect ratio of microholes fabricated with 1, 2, and 4 sub-pulses.

Although Bessel beams can extend the depth of focus, the depth of microholes fabricated by a single Bessel pulse is only approximately 0.4 μm, which is insufficient for achieving high-performance anti-reflective effects. Therefore, MHz burst technology was further introduced to fabricate the microholes (Figure 4a). SEM analysis revealed that the material removal efficiency inside the holes increased significantly with the number of sub-pulses. Figure 4b presents the central cross-sectional profiles of microholes fabricated using 1, 2, and 4 sub-pulses. Compared to the single pulse, the depth of the microholes produced with 4 sub-pulses increased from approximately 0.40 μm to about 0.64 μm, while the aspect ratio improved from approximately 0.3 to 0.7 (Figure 4c,d). By utilizing burst mode to divide the energy into multiple sub-pulses, the plasma shielding effect caused by excessive energy from a single pulse was mitigated. The modulation of the number of sub-pulses in burst mode optimized energy deposition efficiency, enhanced thermal accumulation effects, and facilitated the generation and absorption of free electrons, ultimately leading to an increase in the depth of the microholes [40,41,42,43,44].

**Figure 5:**
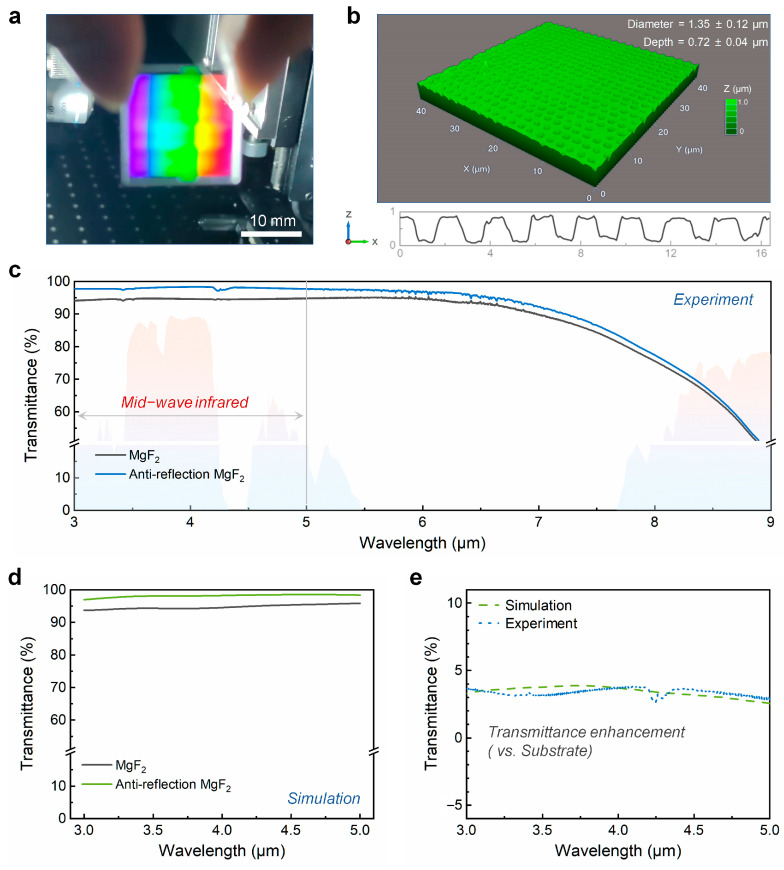
(**a**) Fabricated anti-reflective MgF_2_ window; (**b**) LSCM image of the microholes on the surface of the anti-reflective MgF_2_ window; (**c**) Measured transmittance of the anti-reflective MgF_2_ window (the colored background represents the atmospheric window); (**d**,**e**) Simulated transmittance in the core working band (3–5 μm) of the MgF_2_ window and comparison with experimental testing results.

As a method validation, MHz burst mode femtosecond laser Bessel beams were applied to the fabrication of large-area anti-reflective microhole arrays. Using a laser focal position of 9 μm and a pulse count of 4 (with a laser energy of 491 mW and a repetition frequency of 10 kHz), a 20 mm × 20 mm anti-reflective MgF_2_ window was produced (Figure 5a). Figure 5b shows that the anti-reflective microholes are uniformly distributed with high density across the large area. The diameter and depth of microholes are 1.35 ± 0.12 μm and 0.72 ± 0.04 μm, respectively. The optical performance of the anti-reflective MgF_2_ window was investigated using Fourier transform infrared spectrometer. The transmittance spectra, originally measured in wavenumber by Fourier transform infrared spectrometer, were presented in wavelength for direct relevance to the mid-wave infrared (3–5 µm) band. Compared to the original MgF_2_ window, the transmittance of the MgF_2_ window with dual-sided anti-reflective microstructures showed a significant improvement in the mid-wave infrared range (Figure 5c). Notably, the transmittance at the center wavelength of 4 μm in the mid-wave infrared band increased to approximately 98.3%. Additionally, the transmittance in the core working band (3–5 μm) of the MgF_2_ window was simulated using FDTD Solutions. The theoretical simulation results exhibited a trend in transmittance enhancement that closely aligned with the experimental results (Figure 5d,e). These experimental findings demonstrate that MHz burst mode femtosecond laser Bessel beams can achieve efficient and high-consistency fabrication of large-area anti-reflective microholes. This technique is expected to advance the mass production of anti-reflective windows and enhance the adaptability of infrared thermal imaging in extreme service environments.

## 4. Conclusions

In summary, we have developed a femtosecond laser processing strategy that integrates MHz burst pulse modulation and Bessel beam shaping to achieve high-efficiency and high-consistency fabrication of large-area anti-reflective microholes on MgF_2_ windows. By employing burst pulses with four sub-pulses, the aspect ratio of the microholes was significantly increased from approximately 0.3 to 0.7, while maintaining a high processing speed of 10,000 holes per second. Meanwhile, the use of Bessel beams extended the effective Rayleigh length, enabling a focal tolerance of up to 6 μm while keeping structural deviations below 0.1 μm. Moreover, the Sa and Sq values were 43 ± 3.2 nm and 72 ± 3.5 nm, respectively, demonstrating good stability. This combination effectively addresses the challenges of low processing efficiency and structural inconsistency in the fabrication of high-density microstructures with sub-micrometer feature sizes. As a demonstration, a 20 mm × 20 mm MgF_2_ window was processed with uniformly distributed microhole arrays, leading to a notable increase in transmittance to over 98% in the mid-wave infrared band (3–5 μm). Both experimental and simulation results confirmed the excellent anti-reflective performance of the structured surface. This work provides a practical and scalable approach for the rapid fabrication of functional microstructures over large areas, with potential applications in infrared optics, protective windows, and other devices requiring high transmission and environmental adaptability.

## Data Availability

The data presented in this study are available on request from the corresponding author.

## Supplementary Materials

The following supporting information can be downloaded at: https://www.mdpi.com/article/10.3390/nano15221726/s1, Figure S1: images of a dragonfly’s compound eye and principle of anti-reflection for microstructure arrays; Figure S2: ray diagrams for different incident light wavelengths on the surface of the microstructures.
