# Supplementary material for: Rapid Fabrication of Large-Area Anti-Reflective Microholes Using MHz Burst Mode Femtosecond Laser Bessel Beams
Source: Nanomaterials (Basel). 2025 Nov 15;15(22):1726. doi: 10.3390/nano15221726 (PMC12655269; doi:10.3390/nano15221726)
Supplement: Supplementary file 1 [file nanomaterials-15-01726-s001.zip › nanomaterials-3959028-supplementary.pdf]

Supporting Information

Rapid Fabrication of Large-Area Anti-Reflective Microholes Using MHz Burst Mode Femtosecond Laser Bessel Beams

Yulong Ding, Cong Wang *, Zheng Gao, Xiang Jiang, Shiyu Wang, Xianshi Jia, Linpeng Liu and Ji'an Duan

State Key Laboratory of Precision Manufacturing for Extreme Service Performance, College of Mechanical and Electrical Engineering, Central South University, Changsha 410083, China

Corresponding Authors

*Wang Cong: wangcong@csu.edu.cn

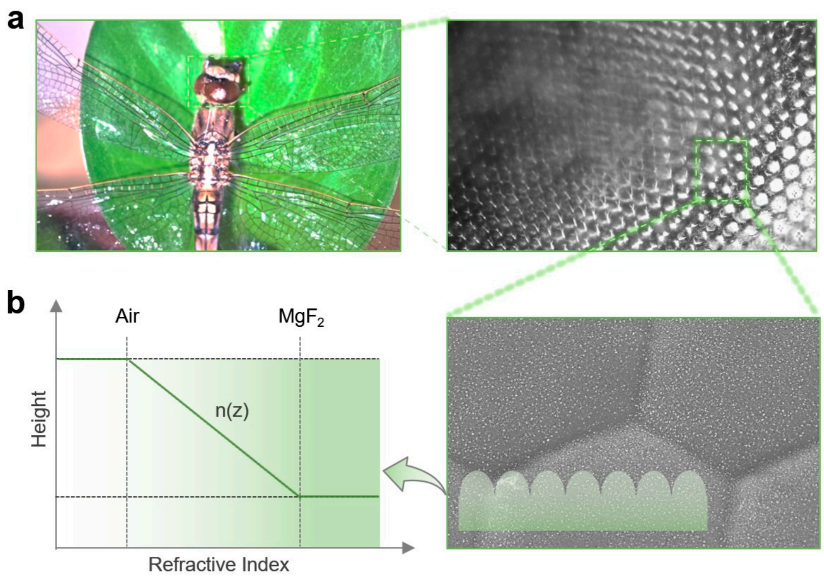

Figure S1. (a) Macroscopic and microscopic images of a dragonfly's compound eye; (b) Principle of anti-reflection for microstructure arrays.

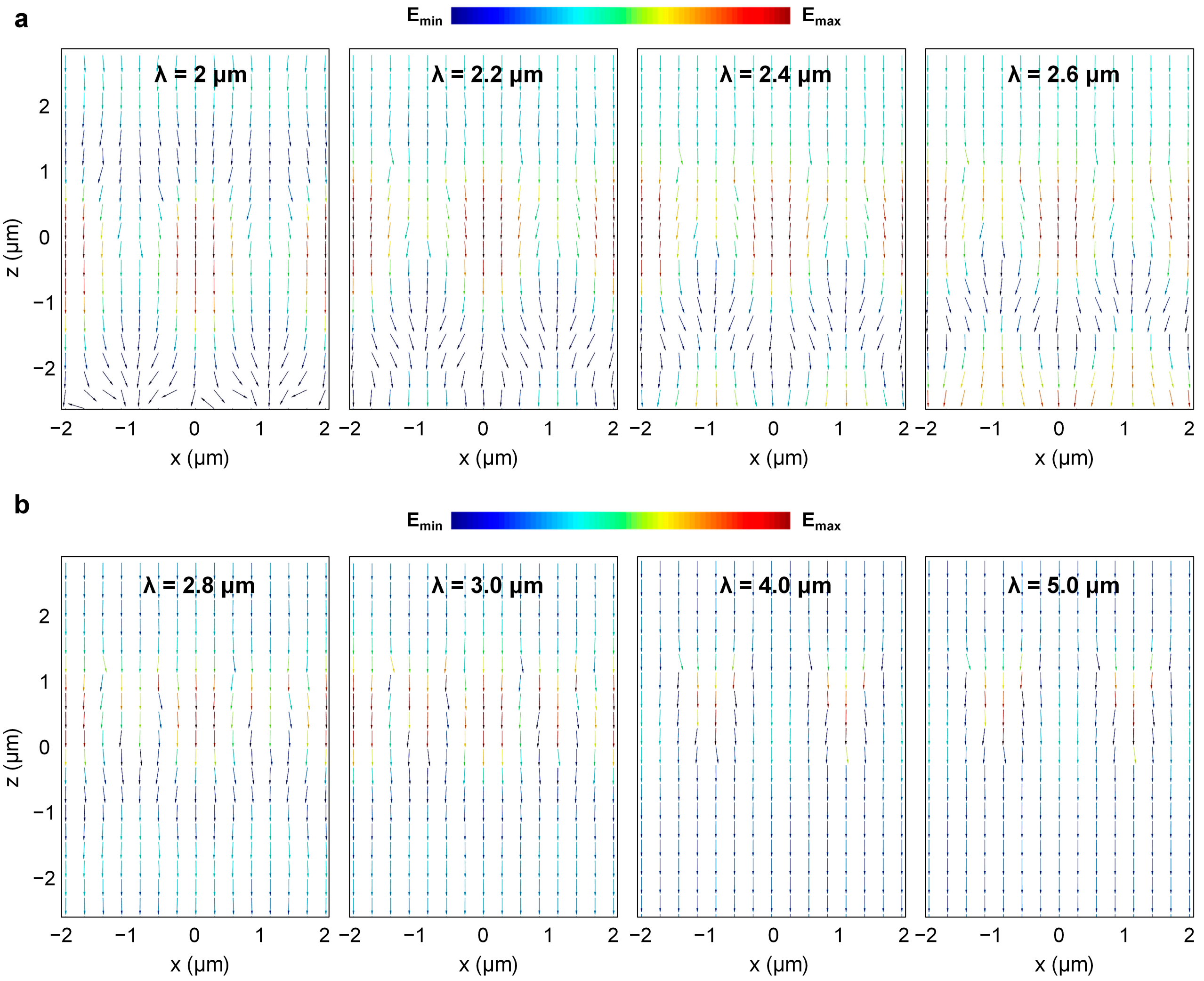

Figure S2. (a) Ray diagrams for different incident light wavelengths (2 μm, 2.2 μm, 2.4 μm, 2.6 μm) on the surface of the microstructures (the structural period and the wavelength do not match, severe scattering and diffraction occur); (b) Ray diagrams for different incident light wavelengths (2.8 μm, 3.0 μm, 4.0 μm, 5.0 μm) on the surface of the microstructures.
